# Retrospective analyses of cisplatin-based doublet combination chemotherapy in patients with advanced gastric cancer

**DOI:** 10.1186/1471-2407-10-583

**Published:** 2010-10-26

**Authors:** Do Hyoung Lim, Se Hoon Park, Keon Woo Park, Jung Hun Kang, Sung Yong Oh, In Gyu Hwang, Jung Mi Kwon, Sang-Cheol Lee, Hui-Young Lee, Hyeong Su Kim, Ho Yeong Lim, Won Ki Kang

**Affiliations:** 1 Division of Hematology-Oncology, Department of Medicine, Sungkyunkwan University School of Medicine, Samsung Medical Center, Seoul, Korea; 2 Division of Hematology-Oncology, Department of Medicine, Dankook University Hospital, Cheonan, South Korea; 3 Department of Internal Medicine, Post-Graduate Medical School, Gyeongsang National University, Korea; 4 Department of internal medicine, Dong-A University College of Medicine, Busan, Korea; 5 Department of Internal Medicine, Chung-Ang University College of Medicine, Seoul, Republic of Korea; 6 Department of Internal Medicine, Jeju National University School of Medicine, Jeju, Korea; 7 Division of Oncology-Hematology Department of Internal Medicine Soonchunhyang University Hospital, Seoul, Korea; 8 Department of Internal Medicine, Kangwon National University Hospital, Kangwon National University School of Medicine, Chuncheon, Korea; 9 Division of Hematology and Oncology, Department of Internal Medicine, Hallym University Medical Center, Hallym University College of Medicine, Seoul, Korea

## Abstract

**Backgrounds:**

Cisplatin-based chemotherapy, in combination with fluoropyrimidines or taxanes, have demonstrated efficacy against advanced gastric cancer (AGC). This retrospective study was performed with the data obtained from our cancer chemotherapy registry and eight another cancer centers.

**Methods:**

In 2008, a total of 283 AGC patients were treated with cisplatin-based doublet chemotherapy in the first-line setting: capecitabine plus cisplatin (XP, n = 77), S-1 plus cisplatin (SP, n = 97), taxanes (docetaxel, paclitaxel) plus cisplatin (TP, n = 72), and 5-fluorouracil plus platinum (FP, n = 37). The primary endpoint of this study was overall survival (OS) and the secondary endpoints were safety, response rate and progression-free survival (PFS).

**Results:**

The median age was 54 years with a range of 28-78 years and median delivered number of chemotherapy cycles were XP: 4, SP: 5, TP: 5 and FP: 5, respectively. Objective tumor responses (38%; 95% CI, 32-43%) were 40% for XP, 42% for SP, 36% for DP, and 24% for FP. The estimated median PFS was 4.5 months (95% CI, 3.6-5.4 months) and the median OS was 12.3 months (95% CI, 10.8-13.7 months). No statistically significant difference was found between each regimen used as first-line chemotherapy. At multivariate analysis, independent prognostic parameters for OS were prior gastrectomy, peritoneal dissemination, performance status and hemoglobin level

**Conclusion:**

All of the cisplatin-based doublet chemotherapy regimens appear to be active as first-line chemotherapy for AGC. With better patient selection according to clinical parameters and molecular markers, clinical outcomes of AGC patients in first-line setting can be improved.

## Background

Gastric cancer is the most frequently occurring malignancy in Korea, and is one of the main causes of cancer death [1]. For patients with recurrent, metastatic, or advanced gastric cancer (AGC), chemotherapy can improve survival, and possibly, provide significant palliation of symptoms [2,3]. Several randomized trials have demonstrated that 5-fluorouracil (5-FU)-based chemotherapy is superior to best supportive care in terms of survival and preservation of quality of life (QOL) [2,3]. While the treatment options for AGC have expanded in recent years to include newer agents such as taxanes (paclitaxel and docetaxel), irinotecan and oxaliplatin, the prognosis of AGC patients remains poor.

Multi-drug combination chemotherapy regimens have generally provided significantly higher response rates, but no better overall survival [4]. Despite the lack of evidence for benefit associated with cisplatin-based combination chemotherapy, it is a common practice to offer cisplatin-based doublet or triplet chemotherapy for AGC patients in the first-line setting, because 5-FU monotherapy has only limited activity. Recently, oral fluoropyrimidines, including capecitabine and S-1, are replacing infusional 5-FU. Both capecitabine and S-1 are considered to be more tolerable than 5-FU, and has been shown to exhibit antitumor activity against AGC [5-7]. Administration of docetaxel, as a component of cisplatin-based doublet or triplet, has also produced significant benefit for AGC patients [8-10]. Although it is currently unclear whether combination of 3 active drugs is superior to doublet combination, our recent randomized study suggested that cisplatin-based doublet and triplet chemotherapy showed similar outcomes [11].

A decision of chemotherapy regimens for an individual patient may be a common clinical situation. Factors considered include the extent of disease, the potential toxicity, especially for those with impaired swallowing or with low performance status, as well as the activity of chemotherapy. Because well-designed, randomized, controlled clinical trials are sparse in AGC, an exploratory, or retrospective analysis seems to be an important source of data to allow the definition of optimal treatment, enhance patient counseling, and generate hypotheses for future studies. We therefore decided to evaluate cisplatin-based combination regimens as first-line chemotherapy for AGC. The present evaluation was also done with the intent to plan and develop improved therapeutic strategies for chemotherapy-naïve AGC patients.

## Methods

This is a multicenter, retrospective study. Between January and December 2008, 283 patients were treated with first-line cisplatin-based doublet combination regimens at 9 tertiary centers in Korea. The criteria for case inclusion were as follows: (1) histologically confirmed diagnosis of gastric adenocarcinoma, (2) no prior chemotherapy or radiotherapy except for adjuvant treatments, (3) presence of metastatic disease, (4) availability of clinical data at the beginning of therapy and follow-up. All patients had been treated with taxane/cisplatin or fluoropyrimidine/cisplatin doublet combination regimens as their first-line therapy for advanced disease. We excluded patients who were enrolled in clinical trials to ensure the choice of chemotherapy regimen was at the discretion of the treating physician. We collected follow-up patient data from the cancer registry. All the data was prospectively recorded and only the survival data was updated at the time of analyses. Written informed consent was given by all patients prior to receiving chemotherapy, according to institutional guidelines. Approval of the study was obtained from the institutional review board or ethics committee.

All patients received cisplatin-based first-line chemotherapy for AGC. Cisplatin was, in all cases, given at a dose of 60-100 mg/m^2 ^infusion on day 1 in combination with capecitabine (1000 mg/m^2 ^bid po on days 1-14, XP; n = 77), S-1 (40 mg/m^2 ^bid po on days 1-21, SP; n = 97), docetaxel (75 mg/m^2 ^iv on day 1, DP; n = 72), or 5-FU (800-1000 mg/m^2^iv on days 1-5 as a protracted continuous infusion, FP; n = 37). Chemotherapy was repeated every 3 weeks (XP, DP and FP) or every 5 weeks (SP) according to the regimen. The treating physician determined chemotherapy regimen, as well as the initial dose of cisplatin, for each patient. Treatment was continued until disease progression or lack of clinical benefit, withdrawal of consent, justifiable withdrawal at the investigator's discretion, or toxicity. Toxicities were graded according the National Cancer Institute (NCI) criteria (CTCAE v3). The dosage of the subsequent cycles was adjusted according to the toxic effects that developed during the preceding cycle. All patients received standard supportive regimen consisting of hydration and antiemetics. The prophylactic use of hematopoietic growth factors was not allowed during treatment, except for patients with febrile neutropenia or grade 4 myelosuppression at the treating physician's discretion. After this combination chemotherapy had failed, second-line chemotherapy was recommended to all the patients if their performance status was preserved. According to the guidelines and department policies, all tumor measurements were assessed after every 2 courses of chemotherapy, by using spiral abdominopelvic computed tomography (CT) scan and other tests that were used initially to stage the tumor. Tumor response was evaluated according to the Response Evaluation Criteria for Solid Tumors (RECIST) [12]. As a general principle for determining clinical response, a confirmatory CT scan was recommended at least 4 weeks apart.

The primary endpoint of this study was overall survival (OS). The starting point of OS and progression-free survival (PFS) was the first day of chemotherapy. The date of disease progression or death from causes other than AGC was used in calculating PFS. Time to death, whatever the cause, was used to calculate OS. PFS and OS were estimated according to the Kaplan-Meier method and the statistical significance of survival curves between groups was tested with a log-rank test. To examine the impact of clinical and treatment variables on the outcomes of chemotherapy, multivariate Cox regression models were used. Covariates included were age (below vs. ≥ median), gender, previous gastrectomy, an Eastern Cooperative Oncology Group (ECOG) performance status (0-1 vs. ≥2), weight loss (>5%) before treatment, number of involved sites (one vs. ≥2), metastases (liver, bone, and bone marrow), presence of ascites, baseline chemistry profiles (serum albumin, alkaline phosphatase, and bilirubin), and chemotherapy regimens. Laboratory parameters were initially recorded as continuous variables and later dichotomized according to the median value of each variable (below vs. ≥ median). All P values were two-sided, with P < 0.05 indicating statistical significance.

## Results

Patient characteristics included in this analysis are shown in Table 1. The median age was 54 years with a range of 28-78 years. Sixty-three percent of patients were male, and 12% had an ECOG performance status of 2. Seventy-three patients had received gastrectomy for curative intent, and 63 patients received adjuvant chemotherapy or chemoradiotherapy. About one-thirds of patients had two or more metastatic disease sites, mostly involving peritoneum and abdominal lymph node. We noted that more DP patients had received adjuvant therapy involving 5-FU, less XP patients had an ECOG performance status of 2, and most of FP patients had peritoneal dissemination at the time of presentation. At the time of data collection, 244 (86%) patients progressed and 177 (63%) were known to have died.

**Table 1:** Patient characteristics

	XP (n = 77)	SP (n = 97)	DP (n = 72)	FP (n = 37)
Age, years				
Median	59	53	51	60
Range	35-77	33-75	28-78	30-75

Gender				
Male	55	63	41	20
Female	22	34	31	17

ECOG performance status				
0	17	15	10	4
1	56	70	51	27
2	4	12	11	6

Prior therapy				
Curative resection	11	22	33	7
Palliative gastrectomy	24	27	16	16
Adjuvant therapy	6	18	31	8

Involved sites(s)				
Abdominal lymph nodes	55	64	39	12
Liver	23	25	16	8
Lung	3	7	3	3
Peritoneum	41	45	39	28
Bone	3	8	9	1
Ovary	4	7	3	1

XP; capecitabine + cisplatin; SP, S-1 + cisplatin; DP, docetaxel + cisplatin; FP, 5-fluorouracil + cisplatin; ECOG, Eastern Cooperative Oncology Group.

A total of 1299 chemotherapy courses were administered. Median numbers of chemotherapy courses for XP, SP, DP and FP were 4, 5, 5 and 5, respectively. The most common reason for treatment discontinuation was disease progression. Overall, cisplatin-based combination chemotherapy was generally well tolerated. We found no relevant difference in the occurrence of grade 3 or 4 toxicities between regimens (Table 2). Two possible treatment-related deaths were identified. One death occurred in the midst of treatment with XP, with no clinical evidence of progression having been demonstrated. Two other deaths occurred in patients while receiving FP, which were attributed to neutropenic sepsis.

**Table 2:** Chemotherapy compliance and toxicity

	XP (n = 77)	SP (n = 97)	DP (n = 72)	FP (n = 37)
Chemotherapy courses				
Median	4	5	5	4
Range	1-9	1-15	1-10	1-14

Reasons for discontinuation				
Progressive disease	28	41	49	21
Toxicity	6	6	4	3
Withdrawal	3	16	3	4
Physician recommendation	34	29	14	9
Unknown	6	5	2	0

Overall grade 3 or 4 toxicity	68	73	67	30

Treatment-related deaths	1	0	0	2

Of a total of 283 patients, 22 could not be evaluated for responses because of the absence of any measurable lesions or early discontinuation of therapy. Objective responses to cisplatin-based chemotherapy were noted in 107 patients (response rate, 38%; 95% confidence interval [CI], 32-43%), including two complete responses seen in XP patients; 40% for XP, 42% for SP, 36% for DP, and 24% for FP. Patients who had a poor performance status (≥2 in ECOG scale) were significantly less likely to respond to first-line chemotherapy (24% vs. 40%; p < 0.001) compared to those with an ECOG performance status of 0 or 1. Other factor associated with lack of optimal response was the presence of peritoneal dissemination (31% vs. 45%; p = 0.047). Response rate was not significantly influenced by age, gender, weight loss, baseline laboratory parameters, metastasis, or chemotherapy regimen.

**Figure 1:**
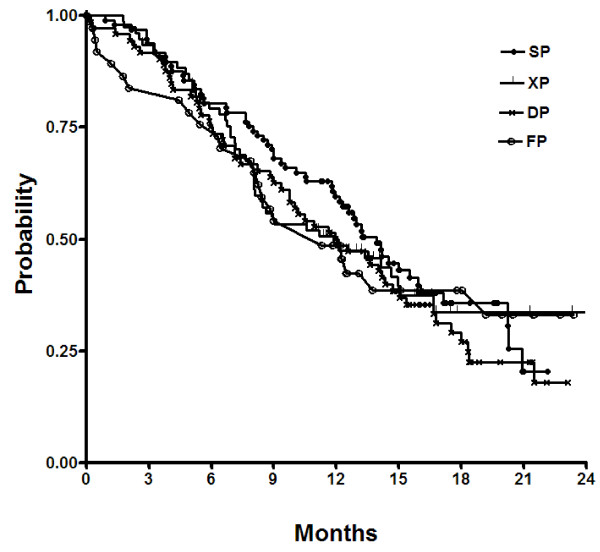
Overall survival according to chemotherapy regimens

The estimated median PFS was 4.5 months (95% CI, 3.6-5.4 months) and the median OS was 12.3 months (95% CI, 10.8-13.7 months). PFS was shorter in patients receiving DP or FP (3.9 months and 3.9 months, respectively) than those receiving XP (5.4 months) or SP (5.7 months). In particular, XP patients had a longer PFS that FP patients (hazard ratio, 0.79; 95% CI, 0.52-1.21; p = 0.29). However, no statistically significant difference in the OS was found between each regimen used as first-line chemotherapy (Figure 1). In the univariate model, the estimated OS was significantly longer for patients with a history of prior gastrectomy, good performance status, normal baseline hemoglobin, single metastatic site, and the absence of peritoneal dissemination (Table 3). Multivariate regression model included the 5 parameters that were found to have prognostic significance in univariate analysis. These parameters were available for all 283 patients. At multivariate analysis, independent prognostic parameters for OS were prior gastrectomy, peritoneal dissemination, performance status and hemoglobin level (Table 4). We also tested whether the OS was modified by interaction between the effect of other significant clinical parameters and chemotherapy regimens given; the first-level interaction term between these variables was entered into separate multivariate model. However, we found no interaction between any first-line chemotherapy regimen with each clinical parameters.

**Table 3:** Univariate analysis according to baseline clinical parameters

		OS, mo*	95% CI	P
Previous gastrectomy	No	10.9	8.8-13.1	.001
	Yes	13.9	12.5-15.4	
Gender	Male	13.3	11.8-14.7	.315
	Female	11.8	9.5-14.2	
Age	<median	11.8	10.3 - 13.4	.279
	≥median	13.4	11.3 - 15.4	
Performance status	0 or 1	13.3	11.9 - 14.6	<.001
	≥2	5.9	1.0 - 10.8	
Hemoglobin	<10 g/dL	7.9	6.0 - 9.8	<.001
	≥10 g/dL	13.4	10.3 - 16.4	
No. of involved site(s)	1	12.8	11.4 - 14.3	.048
	≥2	8.5	6.7 - 10.3	
Liver metastasis	No	12.2	10.4 - 14.1	.654
	Yes	12.2	9.5 - 14.8	
Bone metastasis	No	12.6	10.7 - 14.2	.144
	Yes	8.2	6.5 - 9.5	
Peritoneal dissemination	No	13.9	12.4 - 15.4	.008
	Yes	10.6	8.4 - 12.7	
Weight loss	No	12.6	10.8 - 14.4	.580
	Yes	12.0	9.6 - 14.3	
Chemotherapy	XP	11.2	6.7 - 15.7	.673
	SP	13.3	11.7 - 14.8	
	DP	12.0	8.6 - 15.4	
	FP	11.3	7.1 - 15.5	

* OS, median overall survival; 95% CI, 95% confidence interval.

**Table 4:** Multivariate analysis according to baseline clinical parameters

	P	Hazard ratio	95% CI
No prior gastrectomy	0.002	0.618	0.456-0.836
Two or more metastatic sites	0.347	0.831	0.566-1.222
Peritoneal dissemination	0.013	0.663	0.479-0.916
Low hemoglobin	0.002	0.740	0.610-0.896
Poor performance status	<0.001	0.375	0.245-0.574

After first-line failure, second-line chemotherapy was administered for more than half of patients (n = 162). Specifically, 34 (44%) XP patients received second-line chemotherapy, and 55 (57%) SP patients, 49 (68%) DP patients and 24 (65%) FP patients were treated with second-line therapy. OS was longer in the group able to receive second-line chemotherapy (13.9 vs. 10.1 months, p = 0.024) than in those without further treatment.

## Discussion

Despite recent advances in the treatment of AGC [13], AGC patients treated with first-line chemotherapy have median OS rarely exceeding 12 months. In the current multicenter, retrospective analysis, outcomes for AGC are comparable to those in the published trials of combination chemotherapy. Although this study is retrospective in nature, the results provide a piece of evidence that patients with AGC may derive an indisputable benefit from cisplatin-based doublet chemotherapy.

AGC is an incurable condition where the aim of treatment is to improve survival and to palliate symptoms. Disease may respond to several types of chemotherapy initially, and these treatments have been shown to provide palliation as indicated by improvement in duration and/or quality of survival [2,3]. The best choice of chemotherapy regimen for patients with AGC is still a matter of controversy and requires further investigation [4]. Currently, fluoropyrimidine and cisplatin combination chemotherapy is accepted as a standard regimen by many oncologists. It is presently unclear whether the triplet combination is superior to cisplatin-based doublets for patients with AGC. A meta-analysis showed a difference in OS of approximately 2 months in favor of the anthracycline-containing triplets versus doublets [13]. However, our recently published, randomized phase II study comparing epirubicin, cisplatin plus capecitabine combination with cisplatin plus capecitabine doublet showed similar efficacy outcomes [11]. Among other 3-drug combination regimens, docetaxel-containing triplet chemotherapy (docetaxel, cisplatin and 5-FU) showed superior time-to-progression (5.6 months *vs. *3.7 months) to doublet (cisplatin and 5-FU) [10]. However, the observed OS benefit of triplet was less than one month (9.2 months *vs. *8.6 months), and there was substantial grade 3 or 4 toxicity.

In our analysis, there appears to be no advantage of one cisplatin-based doublet over the others. While there was no relevant difference is OS between treatment groups, it is worth considering the possible role that second-line therapy could have had on survival. More than half of patients received second-line chemotherapy. Furthermore, our observation that PFS was shorter with DP or FP than XP or SP is likely related to negative prognostic factors influencing the choice of intravenous chemotherapeutic agents instead of oral ones. AGC patients who already had peritoneal dissemination, with or without ascites, could not tolerate oral agents. Similarly, we cannot rule out a possibility that the choice of a chemotherapeutic agent depends on previous ones. Although we could not assess time to recurrence after the completion of adjuvant therapy, some AGC patients have received adjuvant chemotherapy with 5-FU, so it seems more prudent to avoid fluoropyrimidines in the first-line regimen for reasons of efficacy. Patients with rapidly progressing disease are an unfavorable setting of patients unlikely to benefit from first-line chemotherapy. For the present, the decision to use specific drug(s) in patients with AGC should be determined by their relative merits on a case-by-case basis.

It is also necessary to better define the sub-population of patients who truly benefit from combination chemotherapy because there is potential for toxicity from the treatment. Therefore, the identification of factors allowing the selection of patients who are likely to benefit from chemotherapy is an important challenge. In a pooled analysis of 3 randomized trials, Chau et al. [14] investigated the prognostic significance of the baseline factors in 1080 chemotherapy-naïve patients with esophagogastric cancer. They found that poor performance status, metastases to liver and peritoneum, and alkaline phosphatase significantly predicted poor survival. Our previous retrospective study in 1455 AGC patients have revealed that poor prognostic factors were no previous gastrectomy, low albumin, high alkaline phosphatase, bone metastasis, the presence of ascites, and a poor performance status [15]. In the current study, hemoglobin level, along with prior gastrectomy, peritoneal dissemination of disease and performance status, emerged as the significant survival predictor. When interpreting the results, it is of note that this analysis represents only a small sample of patients, and we cannot completely exclude the possibility that lower levels of performance status may be reflective of other occult predictors for a poor prognosis.

Although it is conceived that the rationale for offering palliative gastrectomy to patients with unresectable or metastatic gastric cancer is to avoid tumor bleeding, perforation, obstruction, or to improve the outcome by reducing tumor burden, the role of palliative gastrectomy in AGC patients with metastatic disease needs clarification[15]. A randomized trial (reductive gastrectomy for advanced tumor in two Asian countries, REGATTA, KGCA01/JCOG0705) is underway. Besides clinical parameters, appropriate patient selection based on molecular markers is one of the most extensively studied areas in clinical research. While it is still at an early stage and it would take years before we can see clinical application, extensive work is ongoing to identify possible molecular markers, including epidermal growth factor receptor (EGFR) or vascular endothelial growth factor (VEGF) expression [16,17], Her2/neu amplification [18], and excision repair cross-complementing gene 1 (ERCC1) [19], that could be linked to sensitivity or resistance to specific agents, as well as specific genotype variations harbored in different ethnicities. The predictive value of KRAS mutation in metastatic colorectal cancer patients treated with anti-EGFR monoclonal antibodies, cetuximab or panitumumab, has recently been suggested [20]. While gastric cancer is sharing many phenotypic and molecular genetic changes with colorectal cancer, recent molecular studies in gastric cancer have mostly found a lower incidence of KRAS mutation than in colorectal cancer [21,22].

Although cisplatin is often used in combination with other agents, it is well known that cisplatin is associated with significant toxicity and usually requires a high level of clinical monitoring and supportive care including intensive intravenous hydration. Oxaliplatin-based regimens have been actively investigated to improve the efficacy and tolerability of combination chemotherapy for AGC patients[5,23]. Oxaliplatin has significant activity against some cisplatin-resistant tumors and a favorable safety profile over cisplatin[24].

The comparison of the regimens that were the focus of this analysis suggests that cisplatin-based combination chemotherapy may represent a therapeutic ceiling as far as cytotoxic agents are concerned. As seen in a recent phase III study [25], there is clearly interest in exploring molecular markers, and it may be that further advances in the treatment of AGC will only be achieved with development of novel targeted agents. Also, with better patient selection, clinical outcomes of AGC patients in first-line setting can be improved. Emerging science and the knowledge of disease may further guide us to develop individualized treatment for AGC patients.

## Competing interests

The authors declare that they have no competing interests.

## Authors' contributions

DHL and SHP designed the study. WKK provided advice on the study design. DHL, KWP, JHK, SYO, IGH, JMK, SCL, HYL, HSK and HYL followed the patients and collected the data. DHL, WKK and SHP drafted the manuscript. All authors read and approved the final manuscript.

## Pre-publication history

The pre-publication history for this paper can be accessed here:

http://www.biomedcentral.com/1471-2407/10/583/prepub
